# The Merit of Synesthesia for Consciousness Research

**DOI:** 10.3389/fpsyg.2015.01850

**Published:** 2015-12-02

**Authors:** Tessa M. van Leeuwen, Wolf Singer, Danko Nikolić

**Affiliations:** ^1^Department of Neurophysiology, Max Planck Institute for Brain ResearchFrankfurt am Main, Germany; ^2^Ernst Strüngmann Institute for Neuroscience in Cooperation with Max Planck SocietyFrankfurt am Main, Germany; ^3^Centre for Cognition, Donders Institute for Brain, Cognition and Behaviour, Radboud University NijmegenNijmegen, Netherlands; ^4^Frankfurt Institute for Advanced Studies, Johann Wolfgang Goethe UniversityFrankfurt am Main, Germany; ^5^Department of Psychology, University of ZagrebZagreb, Croatia

**Keywords:** synesthesia, consciousness, semantics, parietal cortex, effective connectivity, qualia, NCC, ambiguous stimuli

## Abstract

Synesthesia is a phenomenon in which additional perceptual experiences are elicited by sensory stimuli or cognitive concepts. Synesthetes possess a unique type of phenomenal experiences not directly triggered by sensory stimulation. Therefore, for better understanding of consciousness it is relevant to identify the mental and physiological processes that subserve synesthetic experience. In the present work we suggest several reasons why synesthesia has merit for research on consciousness. We first review the research on the dynamic and rapidly growing field of the studies of synesthesia. We particularly draw attention to the role of semantics in synesthesia, which is important for establishing synesthetic associations in the brain. We then propose that the interplay between semantics and sensory input in synesthesia can be helpful for the study of the neural correlates of consciousness, especially when making use of ambiguous stimuli for inducing synesthesia. Finally, synesthesia-related alterations of brain networks and functional connectivity can be of merit for the study of consciousness.

## Introduction

Synesthesia is a phenomenon in which a presentation of a stimulus, or inducer, produces additional phenomenal experiences, or concurrents, for which no physical sensory inputs exist. For instance, the letter “A” may trigger an experience of red color (Hochel and Milán, 2008) even though there is nothing that can be established as objectively red in the stimulus. Rather, the experience of color red is created exclusively internally. Synesthetic mappings of the inducing stimulus and the concurrent experience are automatic and involuntary, unique for each individual, and generally stable over time (Wollen and Ruggiero, 1983; Baron-Cohen et al., 1987; but see Simner, 2012). The prevalence of synesthesia in the normal population is estimated to be around 1–2% (Simner et al., 2006).

Synesthesia is a unique subject for research particularly because of the additional phenomenal experiences, i.e., the concurrent *qualia*. How and why phenomenal experience arises is part of the “hard” problem in consciousness research (e.g., Chalmers, 1995). Many requirements that are necessary for us to be conscious of the world around us can be explained in terms of functioning, information processing, circuits or systems—for instance, the integration of information by a cognitive system, deliberate control of behavior, and the reportability of mental states (also, among others, named the “easy” problems of consciousness; Chalmers, 1995). This is the relatively easier problem. However, it has turned out much more difficult to explain the mechanisms of *subjective experience* or “what things feel like.” Examples of subjective experiences are for instance the *experience of* seeing red, or the *experience of* feeling the wind on your face on a sunny day. This aspect of consciousness is considered harder to account for.

Many empirical studies have been undertaken to identify neural correlates of conscious experience (Dehaene and Naccache, 2001; Singer, 2001; Melloni et al., 2007; Aru et al., 2012). According to Crick (1994) neural correlates of consciousness is “the minimal set of neuronal events leading to subjective awareness” (Cohen and Dennett, 2011, p.358); Opinions differ widely on how and whether conscious experience can be accounted for by the physiological processes in the brain (Chalmers, 1995; Metzinger, 2000; Gray, 2005; Cohen and Dennett, 2011). For instance, there is no agreement on whether conscious experience and cognitive processing are integrated or not (e.g., Cohen and Dennett, 2011); and on whether conscious experience is established by means of global integration of information (e.g., Lamme, 2006) or by activity in e.g., localized brain areas (Zeki, 2001).

Several researchers have proposed ways in which consciousness research can benefit from the phenomenon of synesthesia. Gray (2005) has argued that synesthesia can provide a window on the hard problem of consciousness. Specifically, Gray proposes that synesthesia can make a case against functionalism—a theory stating that the mental states that constitute consciousness can be defined as interactions between different functional processes. Its prediction, according to Gray, is that for any difference in function, there should be a corresponding difference in subjective experience. The argument put forward by Gray (2005) is that in e.g., colored-hearing synesthesia, two different functions (hearing and color vision) lead to the same subjective experience of color—which is allegedly mediated in brain areas related to color vision. In the meantime, several neuroimaging studies have demonstrated that synesthetic color and veridical color perception do not necessarily share the same neuronal correlates i.e., activated brain regions (Van Leeuwen et al., 2010; Hupé et al., 2012). Moreover, synesthetic colors are often qualitatively different from veridical colors in the sense of texture, quality, and specificity of colors (Eagleman and Goodale, 2009).

Sagiv and Frith (2013) proposed that synesthesia can be used as a model problem for understanding conscious experience for several reasons. Synesthesia is phenomenologically defined while its properties can be studied in detail; there are a wide variety of types of synesthesia providing ample possibilities for testing the neural correlates of various kinds of experiences; synesthetes are normally healthy people and are much more easy to find than for instance patients with specific neurological symptoms. Sagiv et al. suggest that synesthesia should be helpful in the search for the neural correlates of consciousness. In consciousness research it is common to use paradigms in which a subjective change in perception takes place while the stimuli remain constant. Hence, synesthesia can be seen as a special case of changes in subjective experiences that can be contrasted to the conscious percepts of other people in response to the same stimuli. Another interesting point made by Sagiv et al. is with regard to the proposal by Zeki (2001) that consciousness is mediated by “essential (localized) nodes” in the brain that are required for conscious experience. Taking color as an example, synesthesia lends support for that hypothesis because localized differences in activity level of color regions in the brain have been reported for color synesthesia (see e.g., Van Leeuwen et al., 2010; Rouw et al., 2011).

In the present work we suggest several additional reasons why synesthesia has merit for research on consciousness. We first review the research on the dynamic and rapidly growing field of the studies of synesthesia to inform the reader of the current state of affairs. We pay specific attention to the role of semantics in synesthesia, which seems important for establishing synesthetic associations in the brain. We then propose that the interplay between semantics and sensory input in synesthesia can be helpful for the studies of the neural correlates of consciousness, especially when making use of ambiguous stimuli for inducing synesthesia. Finally, alterations of functional connectivity and physical network connectivity discovered in the brains of synesthetes can be useful for the studies of consciousness.

## The role of semantic associations in synesthesia

Synesthesia is a phenomenon that has been known for a long time, with reports dating back as far as 1812 (see Jewanski et al., 2009), but only recently has the phenomenon received due attention from researchers, leading to an explosion in research efforts, largely kicked off by Cytowic's (1993) book “The man who tasted shapes.” The most common tests to determine genuine synesthesia is the test-retest task to assess the consistency of the synesthetic experiences (e.g., Baron-Cohen et al., 1987). In addition, variants of Stroop interference tasks (Stroop, 1935) are often used to provide evidence for the automatic nature of synesthetic associations (Wollen and Ruggiero, 1983; Nikolić et al., 2011). It should be noted that for certain types of synesthesia, the automaticity has also been questioned (Mattingley, 2009; Price and Mattingley, 2013). There are even online resources that enable one to determine whether he or she is a synesthete (e.g., www.synesthete.org; Eagleman et al., 2007). Many different forms of synesthesia exist. Inducers may be letters, words, numbers, time-units (days of week, months), personal names, music, smell, taste, etc… (Day, 2014). However, there are differences in the prevalence of different forms of synesthesia. Just graphemes and time-units may account for 70–80% of all incidences of synesthesia (Day, 2014). There are some very rare forms of synesthesia such as e.g., swimming-style to color synesthesia (Nikolić et al., 2011).

### The nature of inducers

There has been quite a controversy on what the nature of synesthesia really is and how the associations are being created. Before discussing this issue further, it is important to note that little, if anything, is known about how phenomenal experiences may come about in physiological systems. It was therefore difficult for theories of synesthesia to be created on the basis of strong empirical or theoretical foundations. The most straightforward and simple hypothesis was that the additional experiences have to do with additional activation of neurons—presuming rather bluntly, a direct correspondence between elevated firing rates of neurons and phenomenal experience. For example, a neuron coding for red color would be activated and this would then lead, in some unspecified way, to the experience of red. The activation would then come through connections originating from a different brain area—e.g., from the grapheme area to the color area (Ramachandran and Hubbard, 2001). These theories are inspired by the connectionist approach to the brain (McClelland and Rumelhart, 1985), and can be referred to as connection-activation theories (Grossenbacher and Lovelace, 2001; Ramachandran and Hubbard, 2001).

In early days of synesthesia research the theories of aberrant sensory-to-sensory connections were the only ones discussed and studies concentrated on the mechanisms of the hypothetical activation, direct excitation or disinhibition (Grossenbacher and Lovelace, 2001; Ramachandran and Hubbard, 2001). However, later investigations of synesthetic phenomena suggested that the sensory-sensory view of synesthesia should be expanded to allow for *concepts* that can induce synesthesia. It has often been shown that it is not necessarily the sensory inputs that evoke synesthetic concurrents, but rather the extracted meaning of the stimulus. For grapheme-color synesthesia—the most common form and the most frequently studied—this semantic component has been shown reliably and from many different angles (see Chiou and Rich, 2014 for a recent review). For example, one and the same physical stimulus would evoke a different concurrent depending on how the stimulus was interpreted (e.g., the same shape can be understood as an S or as number 5; e.g., Myles et al., 2003; Dixon et al., 2006). Also, it has been shown that new synesthetic associations can be created immediately (within minutes) as new meanings are given to symbols (Mroczko et al., 2009). Indeed, it became soon clear for the most common, and thus most thoroughly studied, forms of synesthesia that they are conceptual in nature (Simner et al., 2006; Novich et al., 2011). Most obvious examples of conceptual synesthesias are days of the week that are colored by their meaning or position in the sequence (Sagiv et al., 2006) as opposed to (or simultaneous with) being colored by the colors of the letter with which the name of the weekday begins; and synesthesias for abstract representations of numerosity such as dice patterns and finger counting—despite the different surface forms, the same number elicits the same color in all cases (Ward and Sagiv, 2007; Ward et al., 2007). Dixon et al. (2000) also demonstrated that synesthesia can occur when synesthetes are merely *thinking* of the inducing stimulus.

These findings of the relevance of semantics were paralleled with failures to replicate the so-called perceptual “pop-out” that certain synesthetes reportedly experience for stimuli that normally do not induce pop-out. Initial case studies had indicated synesthetes experience pop-out (an immediate percept) during visual search (Ramachandran and Hubbard, 2001; Palmeri et al., 2002), but more elaborate (large sample size) studies revealed that this was actually not the case, at least for the majority of synesthetes (Edquist et al., 2006; Sagiv et al., 2006; Gheri et al., 2008; Laeng, 2009; Rothen and Meier, 2009). Synesthetes may show a slight benefit at visual search compared to non-synesthetes (e.g., Palmeri et al., 2002), but for a large majority of synesthetes, synesthesia does not occur pre-attentively (Ward et al., 2010).

These developments had implications for understanding synesthesia. The notion of direct sensory-to-sensory connections as the only mechanism explaining synesthesia had to be revised, requiring researchers to incorporate semantics as a mediator in the process. This has led to the introduction of the term *ideasthesia*, meaning “sensing concepts” (*idea* is Ancient Greek for concept), as a description of the phenomenon (Nikolić, 2009). In essence, ideasthesia is an equivalent to the recently studied phenomena of semantically-mediated crossmodal correspondences (Rubinsten and Henik, 2002; Gallace and Spence, 2006), but, when applied to synesthesia, it generates a specific set of predictions and constraints. Ideasthesia suggests that synesthetes are not born with their associations, as has been suggested earlier, but that the associations have been created by an active process of assigning meaning to a stimulus (to the inducer; Mroczko-Wasowicz and Nikolić, 2014). This happens especially in situations in which synesthetes have difficulties in assigning meaning to stimuli during learning—i.e., a so-called “semantic vacuum.” The theory proposes that synesthetic associations are created to enhance the understanding of the world—to build knowledge. The theory has a strong explanatory power in accounting for the fact that letters, numbers, days of week, and months are the most common inducers; these stimuli are the first abstract concepts that a child is faced with through the educational system. A child has to build a whole new semantic network and a synesthetic child uses synesthesia to enhance this process.

Ideasthesia is consistent with the finding that synesthetes seem to “choose” the concurrent from various sources, before they internalize one of the options and stick with it for a lifetime (e.g., Simner and Bain, 2013). The “choices” for synesthetic concurrents can come from internal and external sources. Many of the crossmodal associations that exist in synesthesia follow patterns of crossmodal associations in non-synesthetes: for instance, the association of high tones with lighter colors and low tones with darker colors (Simner et al., 2005; Ward et al., 2006). Other associations are suggested directly from the external environment, such as the refrigerator-magnet letters (Witthoft and Winawer, 2006, 2013), and others are combinations such as the similarity between shapes of letters that lead to similarities in associated colors (e.g., Brang et al., 2011; Jürgens and Nikolić, 2012), or the sounds of letters (e.g., Mills et al., 2002).

Ideasthesia is not necessarily incompatible with the idea of direct sensory-to-sensory connections in some forms of synesthesias. Relatively noisy and less restricted cortical activation to new (and abstract) stimuli encountered during learning may theoretically lead to random sensory-sensory co-activation in the brain. Synesthetes may be somehow particularly vulnerable or sensitive to such cross-activations (Bargary and Mitchell, 2008; Newell and Mitchell, 2015). During learning, however, semantic processes seem to shape the representation of the (abstract) stimuli and the associated synesthetic experience either becomes incorporated in the higher-level representation of the stimulus, or not (Van Leeuwen, 2014). Newell and Mitchell (2015) propose that although synesthesia may be predisposed in certain individuals, this does not exclude a strong influence of experience and semantics on the final phenotype of synesthesia. Ideasthesia is compatible with this view, but does pose that many forms of synesthesia are strongly dependent on semantics. Note that ideasthesia is not the same as synesthesia without physical input. It is rather the way the physical input is translated into semantics that sets ideasthesia apart.

### Implications for understanding conscious experience

We propose that most of the synesthetic experiences are mediated through semantics. This suggests that also other experiences should be modified by semantics. In fact, Milán et al. (2013) found that cross-modal associations of experiences are not sparse or isolated. Instead, those associations are tightly interconnected into an associative network that closely resembles the semantic network of words. A common example of cross-modal association is a relation of color to temperature (i.e., red is hot, blue is cold) or auditory pitch to visual size (low pitch for large objects and high pitch for small objects; see Spence, 2011 for a review of various crossmodal correspondences). For their study Milán et al. (2013) used Kiki-Bouba shapes (Köhler, 1947) as they found that these shapes have rich personality properties commonly shared among individuals. They conclude that everyday experiences undergo also a form of ideasthesia. This work is closely related to studies of connative meaning (e.g., Walker and Walker, 2012; Walker et al., 2012) in which it is predicted that the same set of cross-sensory correspondences should always emerge regardless of the specific sensory channel (e.g., brightness and high pitch, high pitch and sharpness, etc.) because there is overlap in the semantic interpretation of the stimuli from different modalities. In the framework proposed by Walker et al. (2012) sensory features become linked together conceptually; in Milán et al. (2013) it is also concepts (e.g., personality) that become linked in an associative network.

Research on synesthesia provides a strong case for the role of semantics in phenomenal experience, and this may be helpful in the search for the neural correlates of consciousness. In synesthetes, dependent on context, an ambiguous synesthesia-inducing stimulus (e.g., 5/S) may be accompanied by a different percept. Similar ambiguous stimuli are also available for non-synesthetes such as bi-stable percepts of the Rubin vase/faces or Necker cube; the difference being that in synesthesia, the additional synesthetic percept is *unrelated* to the actual physical sensory input. This suggests possibly a larger separation in the neural representation of the ambiguous stimulus in synesthetes, and thus an easier detection of differential activation with neuroimaging methods in this population. It should be noted, however, that in studying the neural correlates of synesthesia inducing stimuli, both the inducing stimulus and the concurrent synesthetic experience will result in changes in brain activity when the interpretation of the ambiguous stimuli changes. Cross-modality synesthesias might therefore be most suitable for consciousness paradigms, e.g., ambiguous auditory phonemes/words eliciting a color ([ph]/[f], know/no). Moreover, in synesthesia, it has been well established that the change in percept of the ambiguous stimulus is mediated in a top-down fashion—from semantics to the percept. In the case of non-synesthetic ambiguous stimuli, it may be more difficult to disentangle changes in the percept mediated top-down from those occurring through slight differences in bottom-up processing. We therefore propose that synesthesia and especially the role of top-down semantic influences therein can be helpful in determining neural correlates of consciousness, not only with respect to localization but also with respect to the involved networks and possible dynamic interactions between higher and lower level brain areas.

## The physiology of synesthesia

Profiting from non-invasive techniques for recording brain activity and from established research paradigms of cognitive neuroscience, search for the physiological underpinnings of synesthesia has become possible. One of the goals is to discover a physiological difference between the brains of synesthestes and non-synesthetes. Another, arguably even more important, goal is to identify physiological parameters that distinguish between an activated phenomenal experience and a lack of such experience (“neural correlates of consciousness”). In the latter case, the unique properties of synesthetic minds may allow for the needed experimental controls—the presence of an additional experience. If this quest were successful, it may bring us closer to understanding the physiology of phenomenal experience. It should be noted that in synesthesia research, between-group comparisons always bear the problem of individual differences between subjects and the possibility that the synesthete group also differs from non-synesthetes on other aspects (e.g., artistic qualities, mental imagery, or personality, see e.g., Ward et al., 2008; Rouw et al., 2011; Banissy et al., 2013).

### Brain function and structure in synesthetes

Contrasting synesthetic experience with the absence of such an experience, fMRI studies point to excess activity in brain regions that are involved in the processing of the concurrent synesthetic experience. Examples are activity in cortical area V4/V8 when experiencing synesthetic color (e.g., Sperling et al., 2006; Van Leeuwen et al., 2010; for a review, see Rouw et al., 2011), activity in intraparietal sulci for number-form synesthesia (Tang et al., 2008), or excess activity in piriform cortex for olfactory synesthetic concurrents (Chan et al., 2014). The literature is not very consistent, however: For example, not all studies of grapheme-color synesthesia report activity in color area V4 for synesthesia (Rouw et al., 2011). One of the most common findings is excess activity in parietal regions for synesthetes, independent of the specific synesthetic subtype (e.g., Van Leeuwen et al., 2010; Rouw et al., 2011; Neufeld et al., 2012a). Thus, the results may imply a particularly important role of parietal cortex in mediating phenomenal synesthetic experiences.

Electrophysiological studies have demonstrated abnormal processing of synesthesia-inducing stimuli in early processing phases (Beeli et al., 2008; Goller et al., 2009; Brang et al., 2010), over-responsiveness to non-synesthetic parvocellular visual stimuli (Barnett et al., 2008), and abnormal processing of synesthetic concurrent experiences (Van Leeuwen et al., 2013). Several electrophysiological studies suggest that synesthetic effects can occur very early during the processing of the inducing stimuli (105–115 ms, Brang et al., 2010; 100 ms, Goller et al., 2009; 122 ms, Beeli et al., 2008). For synesthesias clearly shown to be based on concepts, such as grapheme-color synesthesia, this would imply that semantics already plays a role early during stimulus processing.

Studies of brain structure have demonstrated both increased white matter and gray matter density (Rouw and Scholte, 2007, 2010; Hänggi et al., 2008; Jäncke et al., 2009; Weiss and Fink, 2009; Banissy et al., 2012; O'Hanlon et al., 2013; Zamm et al., 2013). Regions in which such differences were found are often related to the specific (functional) network of areas involved in the type of synesthesia at hand. For instance, Rouw and Scholte (2007) found increased white matter connectivity in temporal regions close to the grapheme areas and in parietal regions for grapheme-color synesthetes, while Hänggi et al. (2008) found anatomical differences in the auditory and gustatory areas of a tone-interval—taste synesthete. It should be noted that there are also reports of globally altered brain topology in synesthetes (Hänggi et al., 2011). However, it is important to keep in mind that the reports of structural differences in the brains of synesthetes are problematic in the sense that it is not possible to determine—in adult synesthetes—whether the synesthesia is a result of altered anatomy, or whether altered anatomy results from years of synesthetic experience.

### Dynamic connectivity patterns in synesthetes

Altogether, functional and structural neuroimaging studies demonstrate synesthesia-specific alterations in the brain, related to inducing stimuli as well as to concurrent synesthetic experiences. The common theme, however, that emerges from the neuroimaging literature is that communication and connectivity between brain regions appear to be altered in synesthesia. Let us take a look at functional connectivity changes in synesthesia. Changes in functional connectivity patterns in synesthetes have been reported with or without the presence of external stimulation. Tomson et al. (2013) studied networks in grapheme-color synesthetes' brains during rest, auditory grapheme stimulation, and audiovisual grapheme stimulation. Synesthetes had more significant connections during rest and auditory conditions. Investigating the connectivity between 90 anatomical regions, Tomson et al. found that synesthetes showed increased network clustering in visual regions, in line with the type of synesthesia they exhibited. It should be noted that differences in connectivity patterns were also found during rest; this was reported in another resting-state fMRI study as well (Dovern et al., 2012). Dovern et al. found that during rest, connectivity between visual and parietal networks was enhanced for grapheme-color synesthetes, and that the increase in intrinsic network connectivity correlated positively with the strength (consistency) of synesthetic experiences. This work strongly suggests that altered network function is linked to altered conscious phenomenal experiences, even in absence of direct stimulation.

Two more studies have investigated functional connectivity during task performance. In auditory-visual synesthetes, using sounds as stimuli, Neufeld et al. (2012b) found greater connectivity between parietal regions and primary auditory and visual cortices for synesthetes compared to controls. There was no evidence of greater direct connectivity between auditory and visual regions, suggesting a strong role for intermediate areas such as parietal cortex during synesthetic experience. In Sinke et al. (2012), grapheme-color synesthetes showed greater connectivity between parietal regions and early visual cortex (but not the grapheme area). Together these two studies are in line with the resting state results reported above; functional connectivity differences appear to be related to the brain areas that are involved in the specific subtype of synesthesia, with a prominent role for parietal regions. It is important to keep in mind that alterations of functional connectivity can result from either changes in direct physical connectivity or from the alterations in the contribution of the semantic associations.

It is relevant for the study of consciousness—and for studies of the physiology of synesthesia—to consider the strong individual differences commonly detected among synesthetes. Not only do different individuals experience different forms of synesthesia, but also the nature of the concurrent experience can differ (for a review, see van Leeuwen, 2013). One of the most common distinctions in subjective experiences of the concurrent is its spatial location. For instance, in grapheme-color synesthesia the concurrent colors can be experienced either “in the mind's eye,” as if resembling an association, or “projected” into space e.g., located on the same surface as the inducing grapheme (Dixon et al., 2004; Ward et al., 2007). These differences in phenomenal experiences are highly informative for physiological studies on synesthesia: If the phenomenal experiences differ, we can also attempt to find the correlates of these differences in the brain activity or anatomy. To this end, Van Leeuwen et al. (2011) performed an effective-connectivity study, using dynamic causal modeling of fMRI signals. Van Leeuwen et al. demonstrated that the phenomenal experience of the synesthetic colors depends on the direction of information flow between brain areas involved in the phenomenon. For projectors, the data were fit best by a model in which synesthesia modulated the direct influence of the grapheme area onto color area V4. For associators, however, a different model fit the data best—one in which V4 activity was influenced indirectly via higher-order regions. This study demonstrated that the quality of phenomenal experience can depend on the route that information flow takes in the brain, even though the same brain areas are implicated in two different processes.

Two related studies have investigated anatomical changes in the brain in relation to the projector vs. associator status of the subjects. Rouw and Scholte (2007) showed that white matter structural connectivity is generally enhanced in synesthetes. More importantly, they found that white matter changes in temporal regions—those that lay near the so-called grapheme area—were more prominent for projector than for associator synesthetes. In a later study on gray matter structure and function (Rouw and Scholte, 2010), the same authors reported that projector synesthetes had increased gray matter density compared to control subjects in areas generally responsible for perception and action (i.e., visual, auditory, and motor cortex). On the contrary, associators differed from controls in the gray matter density of hippocampus and parahippocampal gyrus, which are regions primarily involved in memory. In conclusion, even relatively subtle individual differences in phenomenal synesthetic experiences can be correlated with identifiable differences in activity and anatomy of brain regions.

## Theories of synesthesia

In the previous section we have discussed that concepts can induce synesthesia—i.e., synesthetic inducers can be of very abstract nature. The question is then, how do the purported physiological mechanisms of synesthesia account for the fact that synesthetic concurrents are shaped by semantic knowledge? Generally speaking, the way we understand the world exerts an influence on how we perceive it (Majid et al., 2004). Apparently, this applies also to synesthetic concurrent experiences. The mechanisms by which synesthesia is mediated by the brain are still being debated and there are roughly two groups of theories. The traditional approaches favor the activation-through-connectivity mechanisms. These presume that connections activate neurons, which then produce the concurrent experiences. The two most important theories in this group are the disinhibition or re-entrant theory (Grossenbacher and Lovelace, 2001; Smilek et al., 2001) and the cross-activation account (Ramachandran and Hubbard, 2001; Brang et al., 2010; Hubbard et al., 2011). Recently, another approach has been proposed based on a new theory of how physiological mechanisms implement semantics (Mroczko-Wasowicz and Nikolić, 2014; Nikolić, 2015). We next discuss each approach in more detail.

According to the disinhibited feedback theory, synesthesia is caused by feedback signals sent from higher-order associative regions to primary sensory regions not originally activated by the inducing stimulus (Grossenbacher and Lovelace, 2001). An example would be the activation of color area V4 via feedback from associative parietal cortex after stimulation of the grapheme area (but not color areas) by a black grapheme. This account of synesthesia allows for context-based and top-down modulation that affects synesthetic experiences via higher-order associative brain regions. The hypothesis implies that inducing stimuli are processed deeply before the conscious synesthetic experience is elicited. This is relevant for our question about the role of semantics in synesthesia: we already know that context can strongly influence synesthetic experiences (e.g., Myles et al., 2003; Dixon et al., 2006). This theory also presumes a significant role for parietal cortex in synesthesia.

The cross-activation theory (Ramachandran and Hubbard, 2001) differs from the disinhibited feedback theory in proposing that activity in the brain regions that are processing the inducing stimulus, directly results in additional activity in brain regions responsible for mediating the concurrent experience. No intermediate, higher-order processing step is included and instead aberrant anatomical connections between the regions processing the inducing and concurrent stimuli are proposed. The lack of an intermediate processing step implies that parietal cortex is not crucial for synesthetic experience: However, in a later update of the cross-activation model, a second stage of (hyper-)binding through parietal mechanisms was added to the theory (Hubbard et al., 2011). In this way the authors acknowledged the growing evidence for an important role of parietal cortex in synesthesia (see below). The cross-activation theory accounts well for fast changes in electrophysiological signals and is consistent with the apparent evidence that synesthetes experience bottom-up perceptual pop-out in serial search task, and with anatomical differences in the brains of synesthetes. However, evidence for pop-out in synesthetes has become challenged over time, and more and more data suggested a crucial role of semantics in shaping synesthetic experiences.

The recent alternative theory of synesthesia proposed by Mroczko-Wasowicz and Nikolić (2014) is based on a novel proposal of how the brain deals with semantics, founded in the theory of practopoieis (Nikolić, 2015). This view implies that the brain can quickly change its computational properties—i.e., it can make quick learning-like changes—and that the extracted meaning of a stimulus reflects those fast changes made to how the network executes its computations. Examples of quick adaptation of computational properties by the brain's network are for instance studies showing that context can affect synesthetic experiences (e.g., an S/5-shaped stimulus presented either in the context of digits of letters; Dixon et al., 2006).

The directionally changed patterns of functional connections in synesthetes found by Van Leeuwen et al. (2011) are partly consistent with the disinhibited feedback account of synesthesia—namely, the model including an indirect pathway to V4 via parietal regions that fit best for the associators—and are partly consistent with the cross-activation theory—namely the model with direct influences between the grapheme and color area that fit best for projector synesthetes. It is clear, however, that the process leading to synesthesia and its related changes in effective connectivity involves a phase of learning abstract entities for which semantics are important. During the phase in which synesthesia develops, the network that processes the inducing stimuli undergoes changes in its computational properties to accomodate the phenomenon of synesthesia. The conscious experience of the synesthetic concurrent is what results; depending on the properties of the established network. Especially relevant here is that conscious experience can depend on the direction of information flow in the network (Van Leeuwen et al., 2011).

## Discussion

The question of why physiological systems have qualia is arguably the most difficult problem faced by neuroscience (Chalmers, 1995; Harnad, 1995). Research on synesthesia, alone, cannot solve the big puzzle of qualia. However, due to the very nature of the phenomenon of synesthesia—i.e., the additional qualia that synesthetes experience—research efforts directed toward understanding that phenomenon may assist in identifying the neural correlates of conscious experience. Thus, we can ask whether the progress made in the last decade or two in the research of synesthesia can be summarized in a way that is informative for consciousness research.

Synesthesia is illustrative for the importance of the extraction of meaning from stimuli for inducing phenomenal experiences. The interplay between the physical synesthesia-inducing stimulus and the way semantic associations finally shape the phenotype of synesthesia helps us to realize that semantics shape our experiences. Experiences do not exist isolated from person's understanding of the world. Semantics and understanding also play a role in the proposal that much of the problems of consciousness are determined by a social reality (Singer, 2015). Importantly, the semantic aspect of synesthesia can also be put to use in the search for the neural correlates of consciousness. Ambiguous stimuli can elicit different synesthetic concurrents of which the neural correlates might be identified. Additionally, because the ambiguity is always resolved by top-down influences, we can investigate the directionality in mediating phenomenal experiences.

On the other hand, physiological investigations of synesthesia have discovered the important role of the parietal cortex (Van Leeuwen et al., 2010; Rouw et al., 2011; Neufeld et al., 2012a) and of relating individual differences in synesthetic experiences to the directions of effective connectivity (Van Leeuwen et al., 2011). Even with activity in similar brain areas, the connectivity between them determines the resulting phenomenal experience. Studies of people with synesthesia under neutral “rest” circumstances also lend support to altered network function that may be directly related to the altered conscious experiences (Dovern et al., 2012; Tomson et al., 2013). These neural correlates of changes in conscious experience are still far away from explaining how physiological mechanisms create experience. Nevertheless, they may present important hints on where to look for explanations and provide constraints for the neural correlates of consciousness.

In summary, synesthesia may inform us about the neural correlates of consciousness because of its unique mix of phenomenal experiences that are largely dictated by semantics, and because of established directionality effects in establishing synesthetic experiences. We hope that consciousness research and synesthesia research will be able to mutually inform each other in the future and that the study of synesthetes will become a mainstream approach in consciousness research.

## Funding

This review was supported by LOEWE-Neuronale Koordination Forschungsschwerpunkt Frankfurt (NeFF).

### Conflict of interest statement

The authors declare that the research was conducted in the absence of any commercial or financial relationships that could be construed as a potential conflict of interest.
